# Retraction: Protective effect of autophagy on endoplasmic reticulum stress induced apoptosis of alveolar epithelial cells in rat models of COPD

**DOI:** 10.1042/BSR-20170803_RET

**Published:** 2021-06-14

**Keywords:** Alveolar epithelial cells, Autophagy, Chronic obstructive pulmonary disease, Endoplasmic reticulum stress

This Retraction follows an Expression of Concern relating to this article previously published by Portland Press.

This article is being retracted from *Bioscience Reports* at the request of the Editor-in-Chief and the Editorial Board following receipt of a notification from a reader, alerting the Editorial Board to the GAPDH Western blots in Figure 4A which are duplicated in several other publications.

The authors were contacted in regards to the retraction and stated that the work was completed by a third party, that the incorrect data was sent to them and that the data is now lost. The authors requested time to obtain new data and correct the article; however, given the extent of the issues raised, the Editorial Board stand by the decision to retract the article.

